# Quantitative characterization of high temperature oxidation using electron tomography and energy-dispersive X-ray spectroscopy

**DOI:** 10.1038/s41598-018-28348-3

**Published:** 2018-07-06

**Authors:** Jihan Zhou, Matthew Taylor, Georgian A. Melinte, Ashwin J. Shahani, Chamila C. Dharmawardhana, Hendrik Heinz, Peter W. Voorhees, John H. Perepezko, Karen Bustillo, Peter Ercius, Jianwei Miao

**Affiliations:** 1 Department of Physics and Astronomy and California NanoSystems Institute, University of California, Los Angeles, CA 90095 USA; 2 Department of Materials Science and Engineering, University of Wisconsin-Madison, Madison, WI 53706 USA; 3 Department of Materials Science and Engineering, University of Michigan, Ann Arbor, MI 48109 USA; 4 Department of Chemical and Biological Engineering, University of Colorado, Boulder, CO 80303 USA; 5 Department of Materials Science and Engineering, Department of Engineering Sciences and Applied Mathematics, Northwestern University, Evanston, IL 60208 USA; 6 National Center for Electron Microscopy, Molecular Foundry, Lawrence Berkeley National Laboratory, Berkeley, CA 94720 USA

## Abstract

We report quantitative characterization of the high temperature oxidation process by using electron tomography and energy-dispersive X-ray spectroscopy. As a proof of principle, we performed 3D imaging of the oxidation layer of a model system (Mo_3_Si) at nanoscale resolution with elemental specificity and probed the oxidation kinetics as a function of the oxidation time and the elevated temperature. Our tomographic reconstructions provide detailed 3D structural information of the surface oxidation layer of the Mo_3_Si system, revealing the evolution of oxidation behavior of Mo_3_Si from early stage to mature stage. Based on the relative rate of oxidation of Mo_3_Si, the volatilization rate of MoO_3_ and reactive molecular dynamics simulations, we propose a model to explain the mechanism of the formation of the porous silica structure during the oxidation process of Mo_3_Si. We expect that this 3D quantitative characterization method can be applied to other material systems to probe their structure-property relationships in different environments.

## Introduction

3D structural analysis is essential to understand the structure and property relationships of materials. One of the important 3D structural analysis techniques is electron tomography, which reconstructs 3D structural information from a tilt series of 2D electron microscopy images^1–3^. Although electron tomography has long been used in the biological sciences^1^, its application in the physical sciences has become more widespread over the last decade^4–12^. More recently, a groundbreaking technique, termed atomic electron tomography (AET)^2^, has been developed to determine the 3D structure of crystal defects and disordered materials such as grain boundaries, stacking faults, the core structure of edge and screw dislocations, point defects and chemical order/disorder at atomic resolution^13–18^. AET has also been applied to localize the 3D coordinates of individual atoms in materials and to correlate crystal defects with material properties at the single atomic level^19,20^. These successes of AET rely on powerful 3D reconstruction methods such as Equal Slope Tomography (EST) and GENeralized Fourier Iterative Reconstruction (GENFIRE)^21–24^. Compared to several popular tomographic methods, it has been demonstrated that EST and GENFIRE can achieve a higher resolution reconstruction from a limited number of projections with a missing wedge^22,24^. Here we apply EST to image the oxidation process of high temperature materials as a function of the oxidation time and the elevated temperature.

High temperature materials play an important role in a wide variety of technological areas, including energy, chemical and aerospace applications^25–29^. In particular there is a drive to find a material to surpass the high temperature limitations of directionally-solidified and single crystal Ni-based superalloys. The high temperature behavior of the Mo-Si-B material system has attracted interest due to its superior creep resistance and oxidation resistance at temperatures above 1200 °C^27,30^. It provides a promising class of potential candidates to replace currently widely used Ni-based superalloys. The oxidation protection of these alloys stems from the continuous outer layer of borosilica glass that develops upon high temperature oxidation exposure^31^. Usually the alloys consist of three phases: Mo in solid solution with Si and B (Mo_ss_), the intermetallic phases of Mo_3_Si (A15) and Mo_5_SiB (T_2_)^27,32^. The composition and phase consistency are important parameters in determining the oxidation response. It has been reported that a sufficient amount of T_2_ phase can improve the oxidation resistance by incorporating B into the silica scale as a fluxing agent, lowering the viscosity to enable flow and ultimately passivation^31^. However, boron additions also act to decrease the toughness and ductility of the alloys. The Mo_ss_ phase is considered crucial for the mechanical properties but offers no intrinsic oxidation protection^27,31^. The oxidation of pure Mo_ss_ causes a linear loss of mass due to continuous sublimation of volatile molybdenum oxides (typified by MoO_3_)^27,33^. To gain an optimal property balance between high temperature mechanical properties and oxidation resistance at high temperatures in Mo-Si-B alloys, it is necessary to design the material based on phase stability and control of microstructure.

In order to understand how the three phases interact with each other during the oxidation of Mo–Si–B alloys, it is necessary to know how they react with oxygen independently of each other. To date, significant efforts have been made in exploring the oxidation process of different phases of Mo-Si-B alloys. Rioult *et al*. performed a comparison between the oxidation of pure Mo, the A15 phase and the T_2_ phase^33,34^. They found that viscous SiO_2_ glass forms a porous, non-protective network of nano-channels after 10 minutes of oxidation at 1100 °C. It is generally believed that the porous network provides a pathway for oxygen diffusion to the substrate, causing continuous oxidation. However, the 3D structure of the oxidized A15 phase has not been studied yet. How the porous network connects and evolves with respect to the oxidation temperature and time remains elusive.

To establish a standard method for quantitative characterization of high temperature oxidation process in 3D, here we applied EST to reconstruct the 3D porous structure of the Mo_3_Si A15 phase formed at varying temperatures and oxidation times and performed a statistical analysis of the pore size. Using energy-dispersive X-ray spectroscopy (EDS), we also conducted an elemental analysis to obtain quantitative chemical information about the formation of MoO_2_. Reactive molecular dynamics (MD) simulations were further employed to understand the formation of the porous structure in Mo_3_Si. On the basis of our 3D reconstructions, we calculated the interfacial shape and normal distributions^35,36^ to quantify the morphology and directionality of our 3D porous structure, respectively. Our results provide new insights into the oxidation process of the Mo_3_Si structure and the formation of the porous SiO_2_ morphology during oxidation.

## Results

### 3D reconstructions of oxidized porous Mo_3_Si structures at elevated temperatures

**Figure 1:**
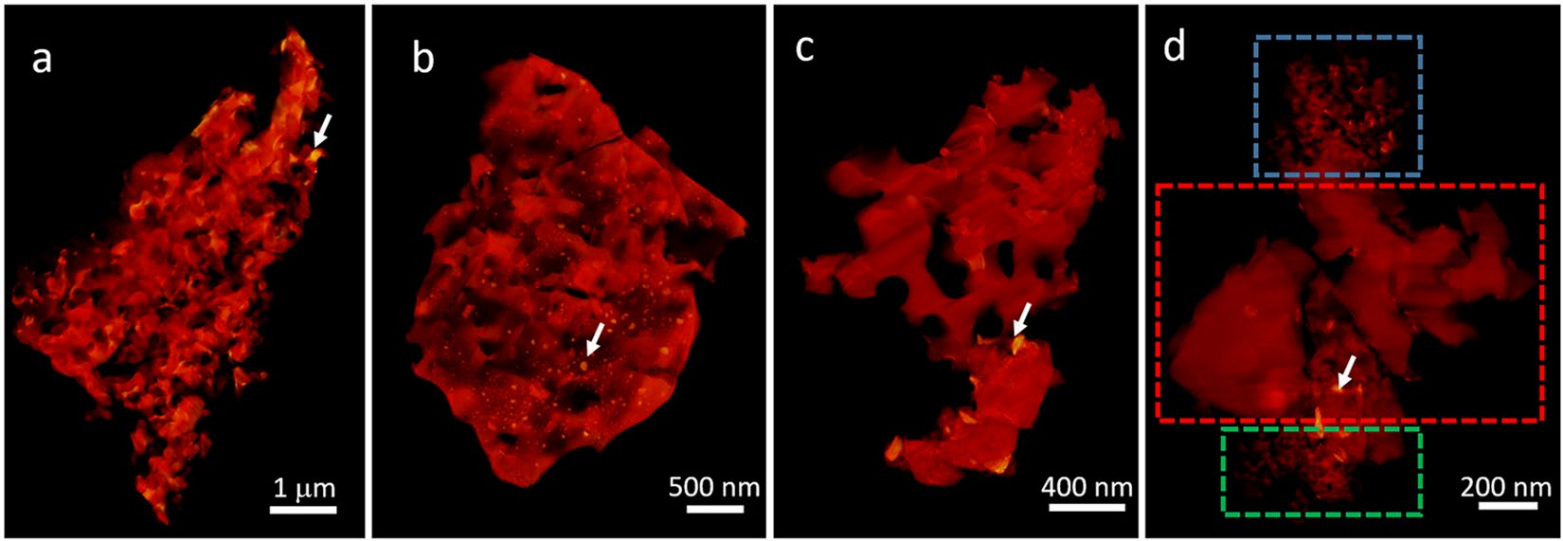
3D volume-renderings of the reconstructed oxidized Mo_3_Si (A15) structures at different oxidation temperatures: 800 °C (**a**), 900 °C (**b**), 1000 °C (**c**) and 1100 °C (**d**). The blue and green squares indicate two highly porous regions with a small pore size (~10 to 30 nm). The red square indicates a region with a large pore size (~100–200 nm). The white arrows show the high intensity regions in the 3D reconstructions.

3D structures were reconstructed from four tilt series of scanning transmission electron microscopy (STEM) images of oxidized porous Mo_3_Si samples, annealed at different temperatures (Methods). Figure 1 shows volume-renderings of the four porous structures at different temperatures after 5 minutes of oxidation in air. The four samples are prepared using the same method but at different temperatures. The pore size is typically around 100–200 nm for all the structures. However, at 1100 °C there are many pores with a diameter as small as ~10 nm and a length as large as ~30 nm, which is about one order of magnitude smaller than the usual pore size. These small pores are also observed in the reconstructions of two other oxidized Mo_3_Si samples at 1100 °C (Supplementary Fig. 1a,b). To gain insight into the mechanism of pore formation, the oxidation of Mo_3_Si was simulated in all-atomic resolution using the reactive MD simulation^37^ (Supplementary Information). The simulations neglect the complex evaporation of MoO_x_ phases for simplicity, and the resulting pores in thermodynamic equilibrium are much smaller than the experimentally measured ones. Also for MD simulations of the oxidation of large Mo_3_Si slabs with size of 20, 40, 100 nm, the SiO_2_ porous structure remains narrow and irregular with the pore diameter between 1 and 2 nm (Supplementary Fig. 2). These results suggest that the formation the mesoporous and macroporous structures in Fig. 1 is mainly due to the evaporation of the gaseous MoO_3_ instead of the oxidation. This process leads to large porous structures far from equilibrium. It has been reported that pure Mo forms MoO_3_ and evaporates rapidly above ~500 °C^38^ leaving only small voids. Our results indicate the high temperature yields small pores (~10 nm) due to volatilizing and the high diffusion rate of MoO_3_ vapor at high temperatures.

**Figure 2:**
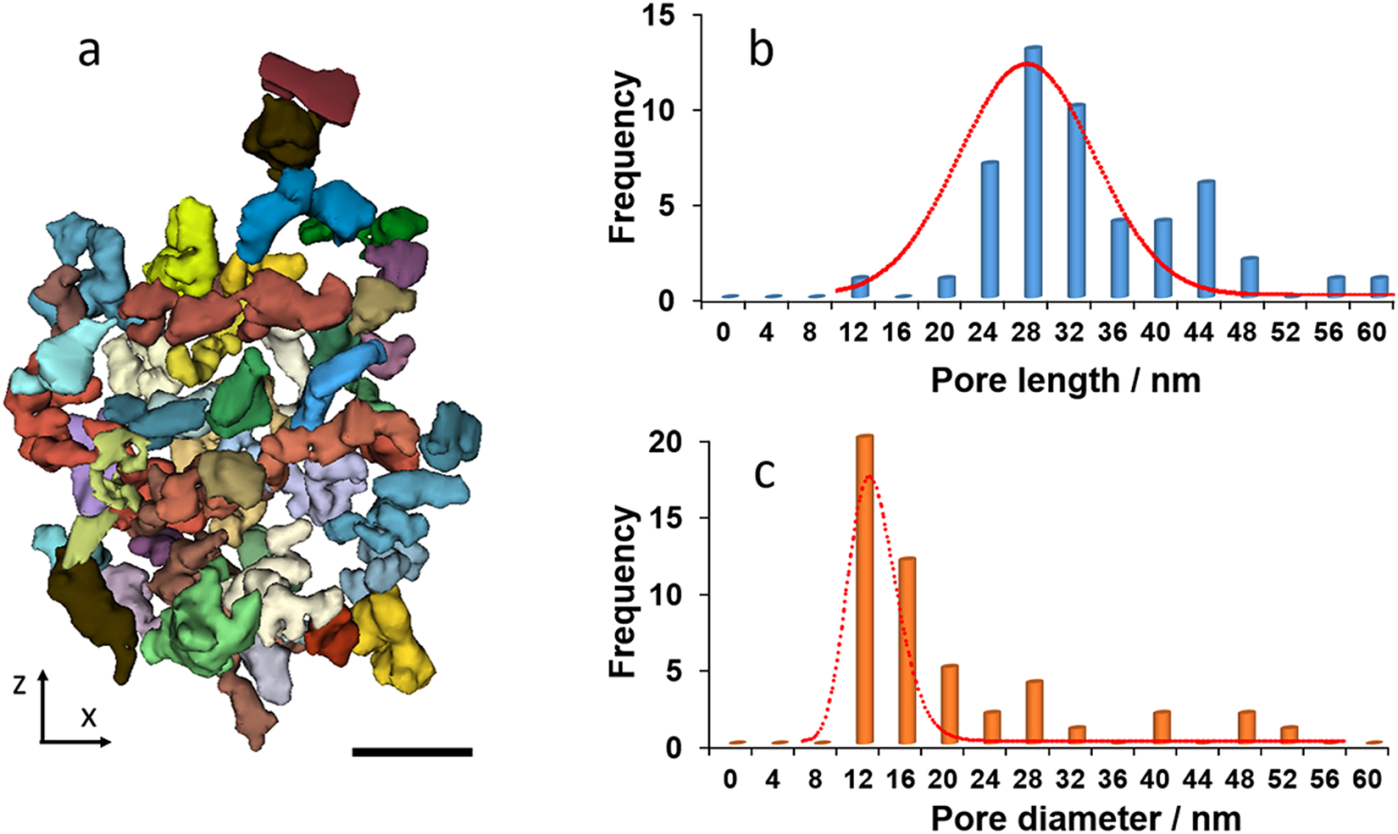
Quantitative analysis of the 3D porous structure. (**a**) Individual pores selected from a highly porous region (the blue square in Fig. 1d). Statistical analysis of the pore length (**b**) and the pore diameter (**c**) distributions for the two highly porous regions (the blue and green squares in Fig. 1d). Scale bar, 100 nm.

The porous structures are highly interconnected (almost ~ 100%) and irregular in shape. The segmentation of these smaller pores was done for the regions with the blue and green squares in Fig. 1d, and the size of the interconnecting points is smaller than 4 nm. The segmentation of the pores was obtained starting from the binarized stack of the initial reconstruction. First the exterior edges of the grain were defined and then a filled mask was applied. The pores were obtained by subtracting the initial binarized stack from the filled image of the grain. As shown in Fig. 2a, each colored isosurface represents a small pore that is separated from other pores by interconnecting points. A statistical analysis of the pore size is shown in Fig. 2b,c. The irregularly shaped pores have an average diameter of 10 nm and a length of 30 nm. Also, we calculated the porosity of these porous structures as 51% and 36% for the blue square region and green square region in Fig. 1d, respectively. We noticed that that the pore size and pore length can vary for different samples. Supplementary Fig. 1c,d show the average pore size and pore length to be 40 and 50 nm, respectively, for two other Mo_3_Si samples oxidized at 1100 °C for 5 min.

### Elemental analysis of porous silica structures with MoO_2_ islands

**Figure 3:**
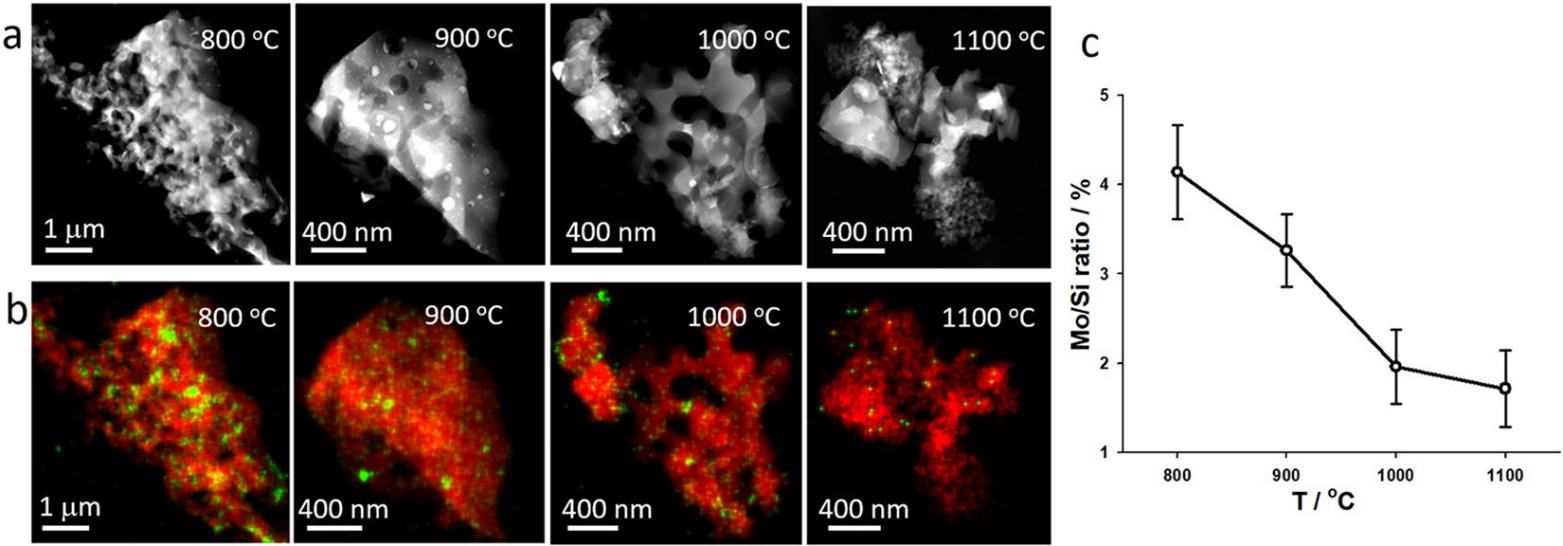
Morphology and composition analysis of porous structures of oxidized Mo_3_Si at different oxidation temperatures. (**a**) HAADF-STEM images at different oxidation temperatures. (**b**) Distribution of Si Kα (red) and Mo Kα (green) in the EDS composition maps of the sample structures in (**a**). (**c**) Quantification of the Mo/Si ratio as a function of the temperature. The error bars were calculated by quantifying several samples at each temperature.

Figure 1 shows 3D reconstructions with very high intensity regions (white arrows), suggesting the segregation of Mo and Si. To test this hypothesis, we collected elemental information from these small high intensity islands within the 3D volume using STEM-EDS. For elemental analysis, high-angle annular dark-field (HAADF) STEM images and STEM-EDS mapping were simultaneously taken at the 0° projection of the same samples used for the electron tomography experiment. Figure 3a,b show the morphology of 3D porous structures and corresponding elemental mappings of Si Kα edge (red) and Mo Kα edge (green), respectively, at different oxidation temperatures. The correlation between the Mo signal in Fig. 3b and the high intensity structures in Fig. 3a indicates that Mo islands were formed in the porous silica structures. We also found that the high exposure temperature yielded a low proportion of the Mo signal.

To determine the Mo to Si ratio as a function of the temperature, we performed a quantitative analysis of the EDS spectra using samples prepared at different temperatures (Supplementary Information, Supplementary Fig. 3 and Supplementary Table 1). Figure 3c shows that the Mo/Si ratio decreases with the increase of the temperatures. From 800 °C to 1100 °C, the Mo/Si ratio decreases by a factor of 2, indicating fewer Mo-rich islands at higher temperatures. HAADF-STEM images show a clear MoO_2_ crystal structure, shown in Supplementary Fig. 4a,b and d. From these images, we calculated the crystal lattice constant to be ~5.5 Å for a and c, which is close to the published value for MoO_2_ (Supplementary Fig. 4c)^39^. Quantification of the EDS spectra from these regions further confirms that these are MoO_2_ but not Mo islands (Supplementary Fig. 5b,c). This observation is consistent with the reported phase equilibria over this temperature range, indicating that MoO_2_ and SiO_2_ are in equilibrium^40^.

### 3D reconstructions of oxidized porous Mo_3_Si with different oxidation times

**Figure 4:**
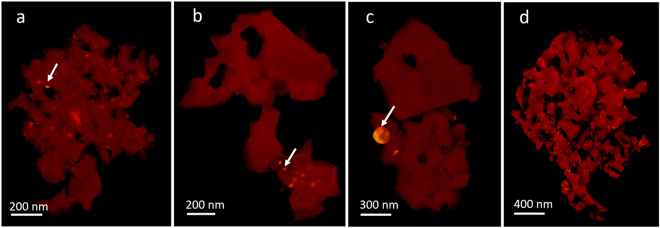
3D reconstructions of oxidized Mo_3_Si with different oxidation times: 5 minutes (**a**), 10 minutes (**b**), 30 minutes (**c**) and 10 hours (**d**). The white arrows indicate the high intensity regions, corresponding to the MoO_2_ islands.

Besides the temperature, the effect of the oxidation time on the porous structures has also been studied. Four samples with different oxidation times were prepared and their 3D structures were reconstructed with EST. Figure 4 shows 3D volume renderings of the porous structures with the oxidation time of 5 min, 10 min, 30 min and 10 hours at 1100 °C in air. After oxidation for 5 min, 10 min and 30 min, MoO_2_ islands can be observed in porous silica (Fig. 4a–c). After 10 hours of oxidation, the whole volume is homogeneous and there is barely any MoO_2_ remaining in the porous regions (Fig. 4d). Although it is difficult to conclude the exact time when all of the MoO_2_ islands disappear, our data indicate that longer oxidation times lead to more complete oxidation and evaporation of Mo_3_Si.

### Morphological Analysis of porous Mo_3_Si structure

**Figure 5:**
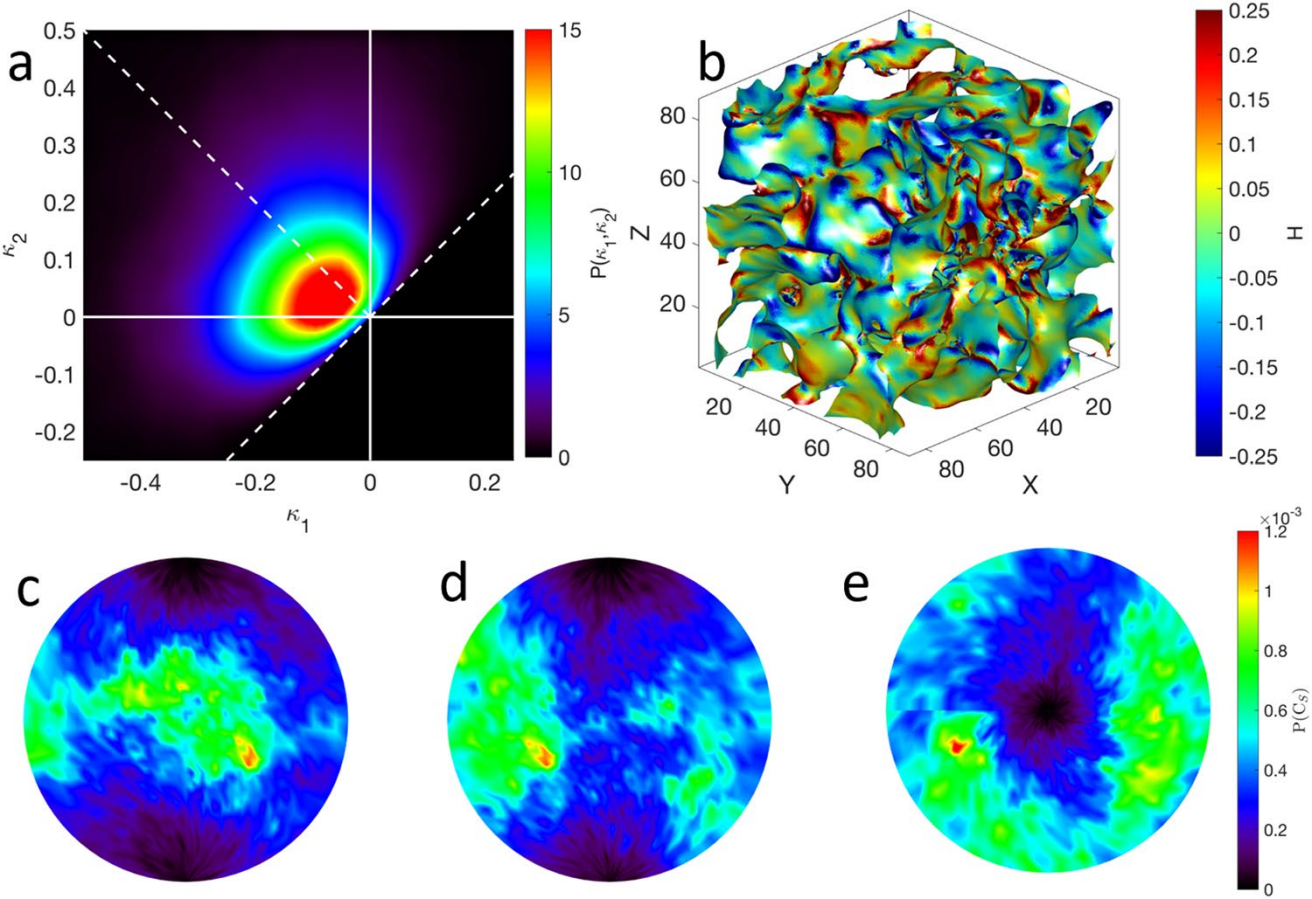
Interfacial shape distribution (**a**) and illuminated mean curvatures (**b**) of porous Mo_3_Si oxidized at 1100 °C. Interfacial normal distributions for porous Mo_3_Si oxidized at 1100 °C for projections along the +x (**c**), +y (**d**), and +z axes (**e**). The area under each IND is normalized to unity. The INDs indicate that the Mo_3_Si structure is isotropic.

We quantified the morphology of a porous region (blue and green regions in Fig. 1d) using the interfacial shape distribution (ISD) method^36^. The ISD gives the probability P of locating an interfacial patch for a range of principal curvatures, ϰ_1_ and ϰ_2_, where ϰ_2_ > ϰ_1_ by definition. The principal curvatures are in turn related to the mean curvature H as H = ½(ϰ_1_ + ϰ_2_). In this classification, interfacial patches have a planar-like shape when ϰ_1_ = ϰ_2_ = 0; a cylindrical-like shape when ϰ_1_ = 0, ϰ_2_ > 0 or ϰ_1_ < 0, ϰ_2_ = 0; and a saddle-like shape when ϰ_1_ < 0 and ϰ_2_ < 0. Figure 5a,b show the ISD and corresponding illuminated mean curvatures of our porous structure oxidized at 1100 °C for 5 min.

We found that the interfacial morphology of porous Mo_3_Si is different from that in another bicontinous structure: nanoporous Au^41^. This suggests a fundamentally different mechanism underlying the formation of the porous Mo_3_Si structure. Nanoporous gold is created by dealloying Au-Ag solid solutions, resulting in open porosity, bicontinuous structures; it yields an ISD that is symmetric about the line ϰ_2_ = −ϰ_1_, corresponding to zero average mean curvature. By contrast, the ISD in Fig. 5a is not symmetric about the line ϰ_2_ = −ϰ_1_. Instead, the ISD indicates a much higher probability of cylindrical-like morphology, along line ϰ_2_ = 0 (H < 0). In Fig. 5b, the preponderance of light and dark blue colors decorating the structure supports this notion that the majority of the interface has a negative mean curvature.

In addition, the directionality of the porous Mo_3_Si structure was obtained by calculating its interfacial normal distribution (IND). The IND gives the probability P of finding an interfacial normal pointing in a given direction. Figure 5c–e. We show the IND using stereographic projections along the +x, +y, and +z axes. The INDs exhibit no peaks or prominent directions. Any variation in INDs is due to noise alone (see color bar). This means the structure is isotropic, as expected for a glass.

## Discussion and Conclusions

The Mo-Si-B alloys have been reported as self-resistant materials from oxidation due to the formation of continuous borosilica glass layer developed upon high temperature exposure. However, there is value in understanding the mechanism of the oxidation process of the single Mo_3_Si A15 phase, as comprehension of the mechanisms at work informs deliberate design.

In evaluating the mechanism, it is instructive to consider the relative importance of the oxidation rate of Mo_3_Si and the volatilization rate of MoO_3_. Specific data for the oxidation rate of Mo_3_Si for the temperature range examined are not available, but a conservative estimation can be made by considering the oxidation behavior of Mo for which data are available. For the oxidation of Mo over the temperature range from 550 to 1700 °C the mass loss rate per unit area, (dM_ox_/dt), is given by

1 $$\frac{d{M}_{ox}}{dt}=A{e}^{-E/RT}$$

where A = 0.504 g/cm^2^s, E = 82.4 kJ/mole, R is the gas constant and T is the temperature^42^. For the oxidation product of MoO_3_, the vapor pressure, P for solid MoO_3_ is represented by

2 $$\mathrm{ln}\,P=\frac{-{\rm{\Delta }}H}{RT}+B$$

**Figure 6:**
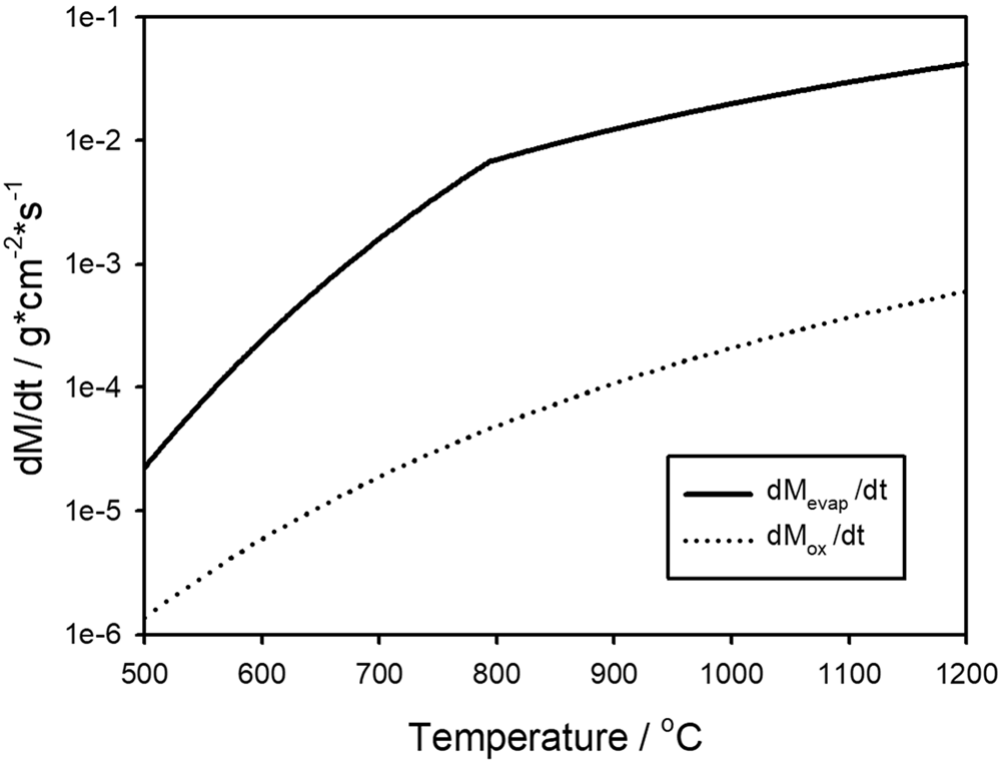
Quantitative comparison between the mass loss from the oxidation of Mo (dashed line) and the evaporation of MoO_3_ (solid line) as a function of the temperature.

Where P is in atm, the enthalpy of sublimation ΔH_s_ = 136056 J/mole, and B = 13.62. Above the melting point of MoO_3_ at 795 °C, the vapor pressure is given by Eq. (2) with ΔH = 63,984 J/mole and B = 5.38^42^. The rate of mass loss per unit area, (dM_evap_/dt) by MoO_3_ evaporation can be evaluated by the Langmuir equation,

3 $$\frac{d{M}_{evap}}{dt}=P\sqrt{\frac{m}{2\pi RT}}$$

where *m* is the molecular weight. For MoO_3_ the dominant vapor species is reported as (MoO_3_)_#_^43^. The comparison of the relative reaction rates is presented in Fig. 6, indicating that the volatilization rate is much greater than the oxidation rate over the entire temperature range. Thus, as soon as MoO_3_ forms it evaporates to expose the substrate to further oxidation and to contribute additional SiO_2_ to the evolving porous silica.

**Figure 7:**
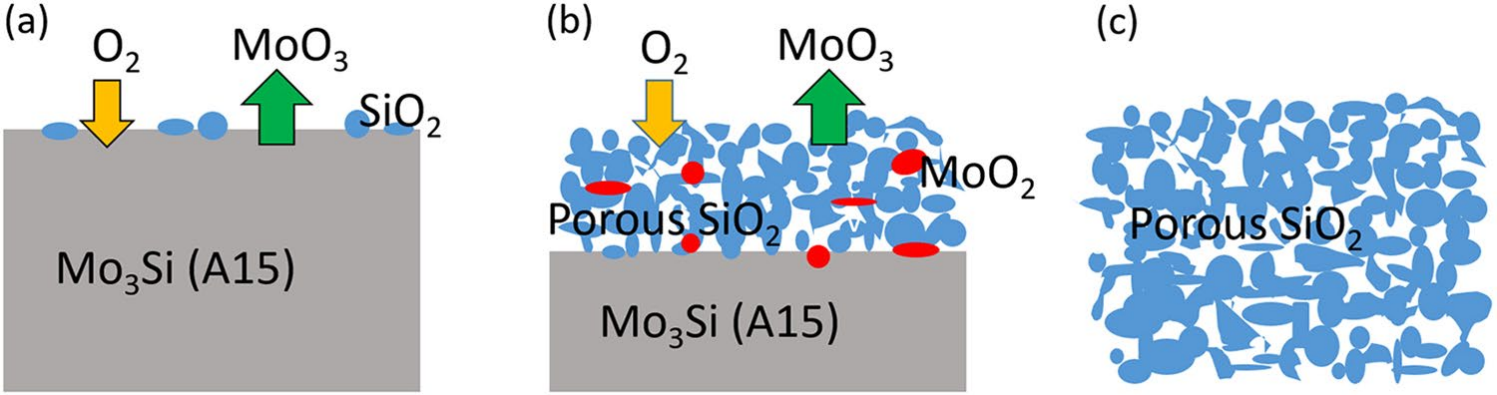
A proposed mechanism for the development of the porous SiO_2_ morphology. (**a**) Exposure of a Mo_3_Si A15 phase to O_2_ at a high temperature results in the formation of SiO_2_ and MoO_x_ and the evaporation of gaseous MoO_3_. (**b**) Porous silica structure starts to develop due to the formation of voids formed after the evaporation MoO_3_. Some MoO_2_ islands also form due to the low O_2_ concentration in silica. (**c**) The Mo_3_Si A15 phase is fully oxidized, accompanying with the depletion of Mo and the complete formation of porous silica.

From the consideration of the relative reaction rates, Fig. 7 shows the whole oxidation process of Mo_3_Si in air. Initially when bulk Mo_3_Si is exposed to oxygen, oxidation begins with formation of small islands of SiO_2_ and evaporation of MoO_3_ (Fig. 7a). Our results have revealed that at an intermediate time during the oxidation there is a porous silica layer with an approximate pore size of 100–200 nm with embedded Mo-rich islands, all on the top of bulk Mo_3_Si (Fig. 7b). The Mo-rich islands are mainly MoO_2_. Previous studies have shown the formation of silica at high temperatures is very viscous and limits the diffusion of oxygen^27,33,34^. As this oxidation proceeds, some regions can eventually reach a sufficient silica thickness to locally lower the partial pressure of oxygen to enable MoO_2_ formation instead of MoO_3_, thus resulting in trapped MoO_2_ in incompletely oxidized specimens. These trapped particles are not protected permanently, however; after exposed to sufficiently high temperature oxidation conditions for a long enough time, we hypothesize that Mo_3_Si will completely oxidize, yielding a pure porous silica structure by depletion of Mo (Fig. 7c).

Using electron tomography and EDS, we obtained the 3D porous structure of oxidized Mo_3_Si at different elevated temperatures and oxidation times. We found that the porous structure highly depends on the oxidation temperature as it changes both the oxidation rate of Mo_3_Si and the evaporation rate of gaseous MoO_3_. We observed that MoO_2_ islands are embedded in porous silica of the oxidation layer. The amount of MoO_2_ decreases as the increase of the temperature. The pore size of silica is typically around 100–200 nm, but much smaller pore size was also observed after 5 minutes of oxidation at 1100 °C. By quantitatively analyzing the porous structure of Mo_3_Si at 1100 °C using ISD methods, we found the pore structure is isotropic, as expected for a glass. The ability to characterize the 3D structure of the oxidation layers in Mo_3_Si A15 phase with elemental specificity is expected to expand our understanding on how the oxidation behavior of Mo_3_Si evolves from early stage to mature stage. It is also clear from our results that the path to improve the oxidation resistance is based upon controlling the viscosity of silica so that it forms a continuous layer rather than a porous structure on the Mo_3_Si phase. Finally, we also want to point out that this quantitative characterization method could be applied to study the oxidation process of other material systems.

## Methods

### Synthesis of Mo_3_Si sample and oxidization process

Mo_3_Si alloy was produced by repeated arc melting of the elemental components in a titanium gettered chamber under Argon. The Mo and Si originate from 99.99% pure elemental reagents. The ingot is melted and re-melted five times to ensure homogeneity, while the ingot mass was measured between each melt to monitor losses. Only ingots with less than 1% loss are accepted. Next the ingot was cut with electrical discharge machining (EDM) into thin slices. These slices are then set onto an alumina boat and loaded into a tube furnace at the exposure temperature (i.e. 800 °C, 900 °C, 1000 °C and 1100 °C) for a certain amount of time (5 min, 10 min, 30 min and 10 hours) in air. After the exposure time, the samples are removed from the furnace to air cool.

### TEM sample preparation and data acquisition

The four Mo_3_Si samples oxidized at 800, 900, 1000 and 1100 °C (typically ~200 μm thickness layer on top of the bulk Mo_3_Si) for 5 min and four Mo_3_Si samples oxidized at 1100 °C for 5 min, 10 min, 30 min and 10 hours were placed in agate mortar and immersed in ethanol, then gently pounded by agate pestle into smaller pieces, separately. The resultant solution was transferred to a plastic tub for sonication of 2 minutes for further dispersion. The supernatant solution was dropped by pipette onto a carbon film on 200 mesh copper transmission electron microscopy (TEM) grids (Ted Pella, Redding, CA). The samples were air-dried for 1 day before data acquisition.

For all TEM experiments, a Titan 60–300 equipped with a HAADF detector (Gatan, Pleasanton, CA) and four windowless silicon drift EDS detectors (FEI super-X) were used with a solid angle of 0.7 srad. The microscope was operated in STEM mode at 200 kV with an electron beam current of ~500 pA for STEM-EDS maps and ~40 pA for HAADF-STEM imaging. A typical total dose for the complete tomography series was ~2600 e^−^/Å^2^ and a typical total dose for an EDS map was ~1000 e^−^/Å^2^. Spectrum images have been acquired with drift-correction over ~120 s assuming a time-dependent linear drift model. For EDS analysis, we used a table of Cliff-Lorimer factors calculated for the 200 kV accelerating voltage to convert the integrated peaks to atomic concentration. The background of each spectrum was subtracted and the Kα of Mo and Kα of Si were fitted by Gaussian models using the Bruker Esprit software. To quantify Mo/Si ratio of porous structures, a fixed stoichiometry for oxides of Mo and Si was specified throughout the whole calculation.

All tilt series of HAADF-STEM projection images were typically acquired between −70° and 70° with a linear tilt step of 1°. The tilt range was limited by the shadowing of the sample holder (Hummingbird, Lacey, WA). HAADF-STEM images were acquired with a convergence semi-angle of 10 mrad at a detector inner semi-angle of 63 mrad and outer semi-angle of 305 mrad. Image size was 1024*1024 pixels with a pixel size from 1.13 nm to 6.47 nm depending on the size of the porous structure. We carefully checked the integrity of the samples before and after taking tomographic tilt series using zero degree projections and before and after EDS mapping, and no sample damage was visible (see e.g. Supplementary Fig. 6).

### Data processing and EST reconstruction

To systematically remove the background, the mean of the background outside of the region of interest (ROI) of each projection was estimated and subtracted for every pixel. A binary mask was then created for the ROI by using thresholding and convolution, whereas the convolution was to maintain the smooth edge of the ROI. The binary mask was applied to the projection and all pixels outside of the mask were set to zero. To balance the signal to noise ratio, the desired resolution and the iterative reconstruction process, 2 × 2 pixel binning was performed for the projection. After background subtraction and binning, all the projections were aligned using the center of mass method^13,15^.

The reconstruction of the aligned projections was conducted using the EST algorithm. During the EST reconstruction, each projection image was assigned to the closest corresponding Fourier plane in the pseudo-polar fast Fourier transform (PPFFT) construct. Most of the angular differences between each measured (constant angular increment) projection and its closest PPFFT plane are less than 0.1°. The small angular difference would have a negligible effect on the 3D reconstruction^22^. The details of the EST algorithm can be found elsewhere^13,15,17,19,21–24^. In particular, it has been shown that EST can increase the spatial resolution and image contrast and reduce the missing wedge effect relative to other tomographic reconstruction methods.

## Electronic supplementary material

Supplementary Information
