# Supplementary material for: Quantitative characterization of high temperature oxidation using electron tomography and energy-dispersive X-ray spectroscopy
Source: Sci Rep. 2018 Jul 6;8:10239. doi: 10.1038/s41598-018-28348-3 (PMC6035195; doi:10.1038/s41598-018-28348-3)
Supplement: Supplementary file 1 — Supplementary Information [file 41598_2018_28348_MOESM1_ESM.pdf]

Supplementary Information

Quantitative characterization of high temperature oxidation using electron tomography and energy-dispersive X-ray spectroscopy

Jihan Zhou[1], Matthew Taylor[2], Georgian A. Melinte[1], Ashwin J. Shahani[3], Chamila C. Dharmawardhana[4], Hendrik Heinz[4], Peter W. Voorhees[5], John H. Perepezko[2], Karen Bustillo[6], Peter Ercius[6], Jianwei Miao[1]

[1]Department of Physics and Astronomy and California NanoSystems Institute, University of California, University of California, Los Angeles, CA, 90095, USA
[2]Department of Materials Science and Engineering, University of Wisconsin-Madison, Madison, WI, 53706, USA
[3]Department of Materials Science and Engineering, University of Michigan, Ann Arbor, MI, 48109, USA
[4]Department of Chemical and Biological Engineering University of Colorado, Boulder, CO, 80303, US.
[5]Department of Materials Science and Engineering, Department of Engineering Sciences and Applied Mathematics, Northwestern University, Evanston, IL, 60208, USA
[6]National Center for Electron Microscopy, Molecular Foundry, Lawrence Berkeley National Laboratory, Berkeley, CA, 94720, USA
Correspondence and requests for materials should be addressed to J.M. (email: miao@physics.ucla.edu)

Porous structure of oxidized Mo_3Si at 1100°C for 5min

Smaller pores are formed at certain oxidation conditions, e.g., for 1100°C 5min, two more reconstructions prepared in different batches show similar small pores comparing to Fig. 1d. The pore length distributions are obtained with the same pore segmentation method.

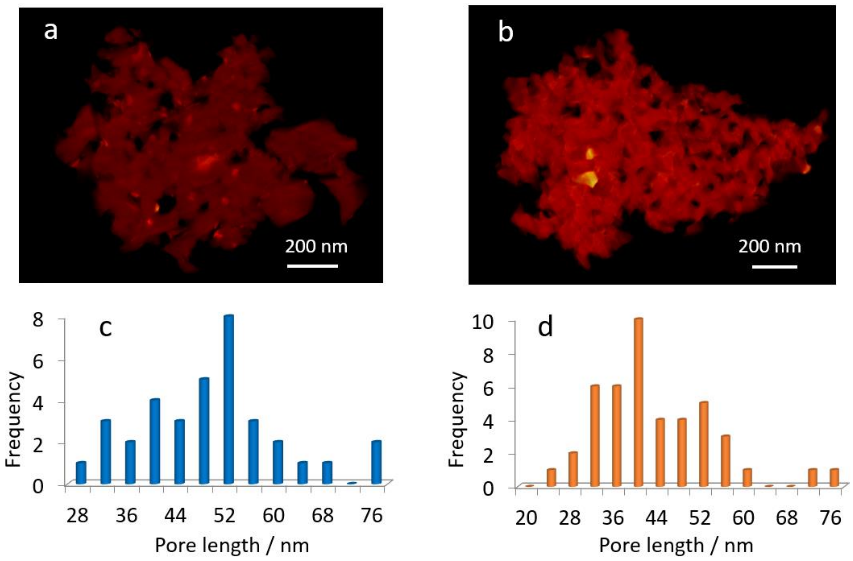

Supplementary Fig. 1. a, b, 3D reconstructions of two porous Mo_3Si samples oxidized at 1100°C for 5 minutes. **c, d**, Statistic analysis of the pore lengths for the two samples shown in (**a**) and (**b**), respectively.

Reactive molecular dynamics simulation of Mo_3Si oxidation

Atomistic models, simulation protocol, and results. The initial model of the Mo_3Si (A15 phase) for reactive molecular dynamics simulations of the oxidation reaction was built using multiples of the unit cell reported in the literature[1]. The Mo_3Si unit cell contains six Mo atoms and two Si atoms. The Si atoms reside in a regular BCC lattice and the Mo atoms on each face represent mutually orthogonal chains (Supplementary Fig. 2a). We employed a small supercell of 12x12x12 (5.8 nm^3) size for initial tests of reactive molecular dynamics, and larger supercells up to $(100\ nm)^3$ size subsequently (Supplementary Figs. 2b-e).

The protocol to simulate the oxidation reaction is as follows. First, we deleted Mo atoms from the 1st surface atomic layer (1 nm) on the (001) surface without further tracking of the chemistry and path of evaporating MoO_3 gas. O atoms were added to the surface Si atoms to match the SiO_2 stoichiometry of the product and atom types of Si and O reassigned to reflect the new chemical identity (see following **Force Field** section). The atomic positions of the bulk alloy beneath the newly forming silica layer were fixed to preserve the Mo_3Si crystal structure and allow free mobility of Si and O atoms. Second, we annealed the silica structure at 1500, 800 and 298 K in the NVT ensemble for 200, 100 and 50 ps respectively, to overcome energy barriers and obtain an equilibrium morphology (lowest energy), where the NVT ensemble means that in the simulations the total number of particles N, the total volume V (solid plus gas phase), and the temperature T were kept constant. Steps one and two were then repeated layer by layer to simulate the successive oxidation of subsequent atomic layers. Thereby, only one layer of atoms in the reaction zone was free to move and all other atoms were fixed (the alloy below and existing silica above the reaction zone). After oxidation of the final number of SiO_2 layers (e.g. 10 layers for the smallest

model), molecular dynamics with mobility of all atoms was performed for 200 ps at 298 K. To test the influence of systems size on the pores formed, simulations were carried out with the initial 5.8 nm model, 20, 40 and 100 nm models using the same simulation protocol. The 100 nm model contained 64 million atoms and rules out any artifacts due to periodic boundary conditions.

Supplementary Fig. 2b shows the oxidation of large (40 nm)[3] Mo_3Si models that feature a 20 nm thick silica layer upon completion of oxidation. Supplementary Figs. 3c, d and e show representative one nanometer thin slices of the silica layer obtained after simulation of the Mo_3Si oxidation reaction from the 5.8, 40 and 100 nm models, respectively. The quantitative analysis of the size and distribution of pores in hundreds of slices indicates irregular pore sizes between 1 and 2 nm with less than 1 nm contact length. The results are independent of the model dimensions and represent the thermodynamic equilibrium state when disregarding the effects of MoO_3 sublimation. Therefore, it can be concluded that the large and irregular pore distribution of the amorphous silica layer on the (001) surface of Mo_3Si observed in experiment is a non-equilibrium structure that results largely from the kinetics of MoO_3 evaporation.

Major limitations of the simulation results are the neglect of MoO_3 evaporation, also called "pesting" process[2], and minor uncertainties from the silica force field (see next section). The simulation protocol of the reaction relies on the assumption of a layer-by-layer process. While there is no direct evidence, an interval in between oxidation of subsequent layers allows better qualitative comparisons to the long time scales (seconds) in experiment. The reported results do not depend on the layer thickness, verified by tests with larger incremental layer thickness of 2 nm. The simulation protocol also keeps computational cost at a manageable level, especially for the large-scale simulations up to 100 nm size.

Force Field. A new force field for bulk silica was created for the reactive simulation of the oxidation of the Mo_3Si (001) surface. The potential energy expression consist only of nonbonded parameters to allow changes in chemical bonding, including a term for Coulomb energy and a 12-6 Lennard-Jones (LJ) term, $E_{pot} = E_{coulomb} + E_{LJ}$. The parameterization is based on the INTERFACE force field approach[3] and higher atomic charges account for the neglect of covalent bonding in silica. This force field is also compatible with other common force fields such as CHARMM, CVFF, and AMBER for interfacial simulations[4,5]. The parameters reproduce the local tetrahedral geometry, Si-O bond length, and density of crystalline silica phases (quartz, cristobalite). In total, four different atom types were defined including two for Mo and Si in the alloy phase as well as two for Si and O in silica. Mo and Si in the alloy carry no atomic charges (zero) and are described by LJ parameters (σ, ε) only which amount to (2.99 Å, 5.0 kcal/mol) and (4.55 Å, 3.0 kcal/mol), respectively. Si and O atoms in silica carry atomic charges of +2.4e and -1.2e, respectively, as well as LJ parameters of (1.80 Å, 0.3 kcal/mol) for Si and (3.47 Å, 0.027 kcal/mol) for O.

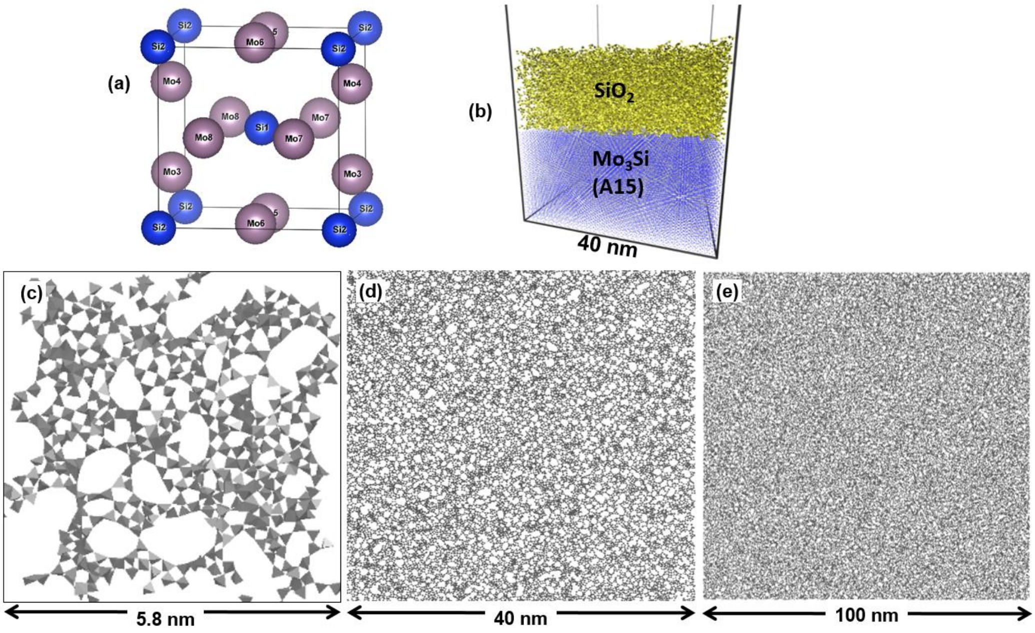

Supplementary Fig. 2. The unit cell of Mo_3Si and results from reactive molecular dynamics simulation of the oxidation reaction of the (001) surface of Mo_3Si, neglecting MoO_x evaporation. The formation of an amorphous SiO_2 layer with only small pore size can be seen. **a**, Unit cell of Mo_3Si (A15 phase). Silicon atoms are shown in blue, molybdenum atoms in dark orchid color. **b**, Morphology of a 20 nm thick layer of silica after oxidation of a $(40\ nm)^3$ Mo_3Si slab. **c-e**, 2D representation of the silica morphology of 1 nm thin slices in top view after oxidation (silica tetrahedral shown in gray), obtained from the 5.8, 40 and 100 nm models, respectively. The pore size is between 1 and 2 nm and distances between pores are of equally small distance. The structures correspond to an equilibrium morphology in the absence of kinetic effects from MoO_x evaporation. For more detailed MD results, please refer to ref. 6.

EDS and high-resolution images of MoO_2 islands

To get statistical results, except for the 0° projection EDS maps of flakes characterized by electron tomography, around 5 more spectra of similar flakes oxidized at each temperature were also collected and then averaged for Fig. 3c. One typical fitted EDS spectrum of Mo and Si are shown in Supplementary Figs. 3a and 3b. and the raw spectrum is shown in Supplementary Fig. 3c.

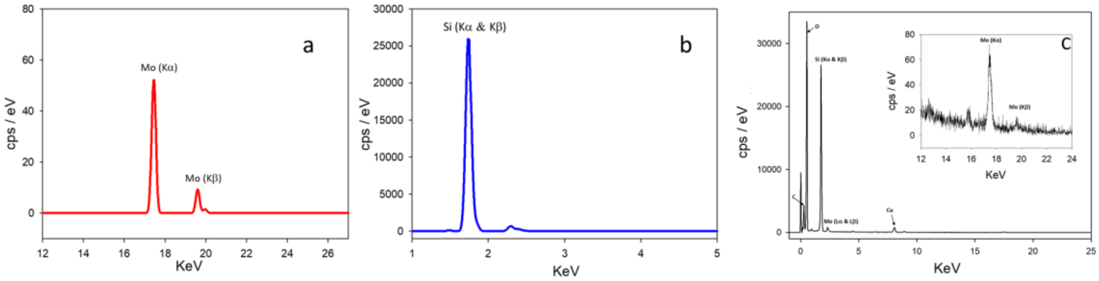

Supplementary Fig. 3. Quantification of Mo/Si ratio. Fitted spectra of Mo (**a**) and Si

(**b**) from an EDS map. **c**, An EDS spectrum of a Mo_3Si porous structure. The Cu and C signals are from the TEM grid. The oxidation temperature was 900°C.

We used a table of Cliff-Lorimer factors calculated for 200kV accelerating voltage to convert the integrated peaks to atomic concentration using Bruker Software. We used the K-edges of all elements, because the scattering cross-sections of the K-edge are better known. Consequently, the calculated CL factors are more accurate. As we do statistical analysis for multiple flakes at the same oxidation temperature, the error bar for Mo/Si ratio comes from the results of analysis of 5 EDS maps of different sample flakes. The magnitude of the error bar is usually ±1% (~15 to 20% of error). To confirm the analysis without an absorption correction is not a problem for our results, we repeated the quantification using an absorption correction and found the change in mole fraction to be less than 10 relative % of the reported values for both Si and Mo. For the density we used the density of non-porous SiO_2 (2.7 g/cm^{-3}), which is the dominant phase. We quantified the spectra using a thickness of 50, 200 and 500 nm which spans the extremes of the thickness of our flakes. Results for one spectrum of one sample oxidized at 800°C for 5min are shown in Supplementary Table 1 below. The difference between these two extremes (50 and 500 nm) was smaller than the error bars in quantifying different flakes. We therefore conclude that the absorption correction is negligible; this makes sense considering both the low Z of most of the material as well as the fact that it is porous. We fixed the stoichiometry of the oxides to be dioxides to get a more accurate result, as SiO_2 and MoO_2 are the most likely oxides we have in the porous structure during high temperature oxidation[6]. We also calculate the results if we don't assume fixed stoichiometry. The result for Mo/Si ratio is within 5% accuracy between the results with and without fixed stoichiometry. The oxide is $MO_{1.7}$ (M stands for Si or Mo) without fixed stoichiometry without absorption correction. The lower estimation of oxygen is due to the absorption.

Supplementary Table 1. Thickness effect on the Mo/Si ratio when considering an absorption correction

Thickness / nm	Mo mole fraction	Si mole fraction	Mo/Si ratio
50	4.54%	95.46%	4.76%
200	4.40%	95.60%	4.60%
500	4.14%	95.86%	4.32%

To confirm the structure of Mo oxide islands we observed in the 3D reconstruction and EDS mapping, high resolution images of Mo oxide were taken and shown in Supplementary Fig. 4. <001> and <010> crystal orientations of MoO_2 are shown in Supplementary Figs.4a and 4b respectively. We also calculated the crystal lattice constant based on the HR-STEM images measurement (Supplementary Fig. 4d), and it is ~5.5 Å for both a and c which is close to the literature published[7] (5.54 Å for a and 5.63 Å for c, Supplementary Fig. 4c). Supplementary Fig. 5 shows the overlay of Mo, Si and O signal obtained from EDS mapping. Clearly, Mo signal is associated

with O signal while no Si signal can be seen at the same region (white circles in Supplementary Fig. 5b). The oxygen peak can be seen from these regions in EDS spectrum (Supplementary Fig. 5c). It indicates the Mo forms MoO_2 embedded in silica.

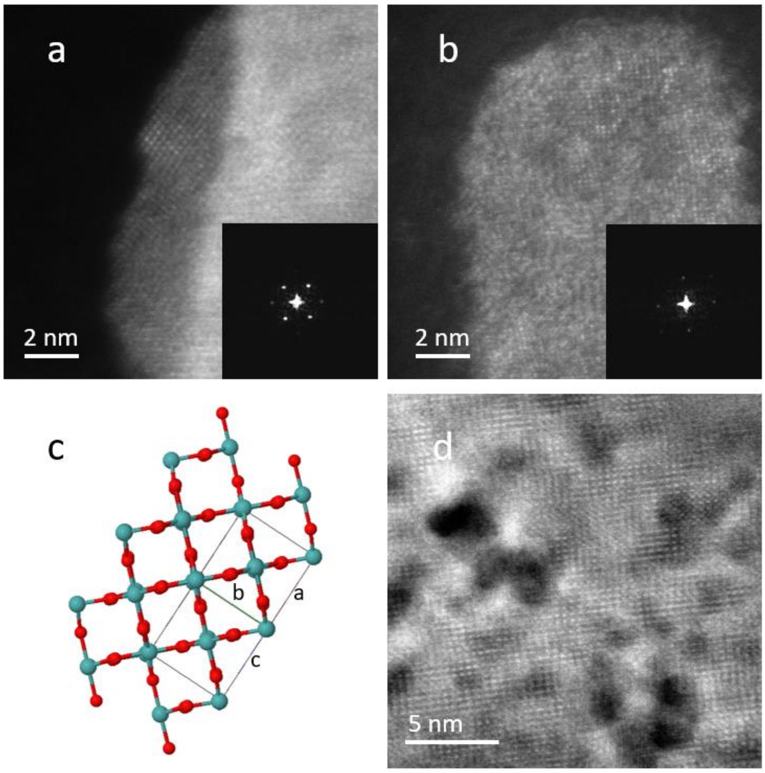

Supplementary Fig. 4. a, b, High resolution STEM images of MoO_2 lattice. The insets show the Fourier transform of the lattice structure. **c**, a 3D atomic model of MoO_2. **d**, A STEM image of a MoO_2 sample at the same crystal orientation as in (**c**).

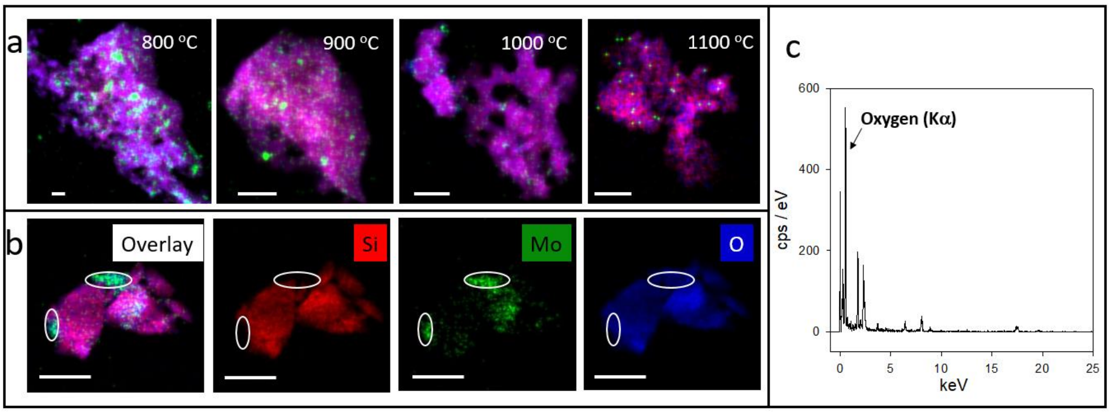

Supplementary Fig. 5. a, EDS maps of the four oxidized Mo_3Si samples shown in Fig. 3. **b**, Representative elemental mapping for Si, Mo and O. The white ellipse indicate the MoO_2 regions. **c**, EDS spectrum of the Mo-rich regions indicated by white ellipse (**b**), where a strong O peak is visible. The EDS spectra of several other samples show the similar O peak. The oxidation temperature for (**b**) and (**c**) was 1000 ^{o}C

Stability test of the samples before and after tomography and EDS mapping

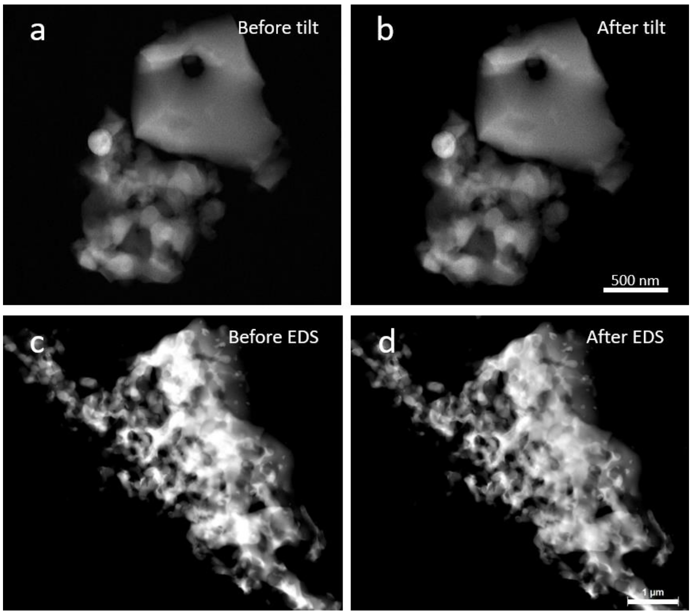

Supplementary Fig. 6. STEM images at 0° before (**a**) and after (**b**) acquiring a full tomographic tilt series of a sample oxidized at 1100 °C for 30 minutes. The 3D reconstruction of the tilt series is shown in Fig. 4c. STEM images before (**c**) and after (**d**) measuring an EDS map of a sample oxidized at 800 for 5 minutes. The EDS map is shown in Fig. 3.

References

1. Templeton D. H. & Dauben, C. H. The crystal structure of Mo_3Si. *Acta Crystallogr.* **3,** 261-262 (1950).
2. Zhang, D. Z. S. *Aerospace Materials Handbook.* 254 (CRC Press 2012).
3. Heinz, H., Lin, T.-J., Mishra, R. K. & Emami, F. S. Thermodynamically consistent force fields for the assembly of inorganic, organic, and biological nanostructures: the interface force field. *Langmuir* **29,** 1754-1765 (2013).
4. Heinz, H., Koerner, H., Anderson, K. L., Vaia, R. A. & Farmer, B. L. Force field for Mica-Type silicates and dynamics of octadecylammonium chains grafted to montmorillonite. *Chem. Mater*. **17,** 5658-5669 (2005).
5. Emami, F. S. *et al.* Force field and a surface model database for silica to simulate interfacial properties in atomic resolution. *Chem. Mater*. **26,** 2647-2658 (2014).
6. Dharmawardhana, C. C. *et al.* Reliable computational design of biological and inorganic materials to the large nanometer scale using the interface force field. *Mol. Simul*. **43,** 1394-1405 (2017).
7. Villars, P. (Chief Editor), MoO_2 Crystal Structure. *PAULING FILE in: Inorganic Solid Phases, SpringerMaterials (online database), Springer, Heidelberg (ed.) SpringerMaterials* http://materials.springer.com/isp/crystallographic/docs/sd_1503100 (2016)
